# Evaluation of Quality of Life at Progression in Patients with Soft Tissue Sarcoma

**DOI:** 10.1155/2017/2372135

**Published:** 2017-04-23

**Authors:** Stacie Hudgens, Anna Forsythe, Ilias Kontoudis, David D'Adamo, Ashley Bird, Hans Gelderblom

**Affiliations:** ^1^Clinical Outcomes Solutions, Tucson, AZ, USA; ^2^Purple Squirrel Economics, New York, NY, USA; ^3^Eisai Ltd., Hertfordshire, UK; ^4^UT Southwestern Medical Center, Dallas, TX, USA; ^5^Department of Medical Oncology, Leiden University Medical Center, Leiden, Netherlands

## Abstract

*Introduction*. Soft Tissue Sarcoma (STS) is a rare malignancy of mesodermal tissue, with international incidence estimates between 1.8 and 5 per 100,000 per year. Understanding quality of life (QoL) and the detrimental impact of disease progression is critical for long-term care and survival.* Objectives*. The primary objective was to explore the relationship between disease progression and health-related quality of life (HRQoL) using data from Eisai's study (E7389-G000-309).* Methods*. This was a 1 : 1 randomized, open-label, multicenter, Phase 3 study comparing the efficacy and safety of eribulin versus dacarbazine in patients with advanced STS. The QoL analysis was conducted for the baseline and progression populations using the European Organization for Research and Treatment of Cancer 30-item core QoL questionnaire (EORTC QLQ-C30).* Results*. There were no statistical differences between the two treatment arms at baseline for any domain (*p* > 0.05; *n* = 452). Of the 399 patients who experienced disease progression (unadjusted and adjusting for histology), dacarbazine patients had significantly lower Global Health Status, Physical Functioning scores, and significantly worse Nausea and Vomiting, Insomnia, and Appetite Loss (*p* < 0.05).* Conclusions*. These results indicate differences in HRQoL overall and at progression between dacarbazine and eribulin patients, with increases in symptom severity observed among dacarbazine patients.

## 1. Introduction

Soft Tissue Sarcoma (STS) is a rare malignancy of mesodermal tissue, with international incidence estimates between 1.8 and 5 per 100,000 per year [1]. Data from the Surveillance Epidemiology and End Results (SEER) database suggest that, despite low overall incidence, incidence is positively correlated with increases in age and is estimated to be as high as 18.2 cases per 100,00 among adults over the age of 70.2 years. Patients with STS account for approximately 0.7% of all new cancer cases and roughly 0.8% of all cancer deaths [2]. The rate of new STS diagnoses has increased steadily over time, with an average yearly increase of 1.8% between 2002 and 2012 [2]. The American Cancer Society estimates that 12,310 new cases of STS will be diagnosed in the United States (US) in 2016.

The 5-year overall survival estimate for STS is 64.9%, though this varies considerably between various staging levels. Exactly 81.4% of patients diagnosed with localized STS survive to year 5 compared to 17.3% among those diagnosed with metastatic STS. A 20-year longitudinal study discovered that 5-year survival rates increased by 28% in the period from 1992 to 2012, with improved detection and efficacy of radiotherapy listed as potential sources of the improvement [3].

Surgery is usually the initial management strategy for localized STS. Postoperative radiotherapy is encouraged in the National Comprehensive Cancer Network treatment guidelines for patients with STS to limit the rate of local recurrence and improve the progression-free survival (PFS). Even in cases in which optimal localized treatment was achieved, distant metastases occurred in many patients with STS, especially those with high-grade tumors. Studies examining the effect of adjuvant chemotherapy have produced equivocal results, and despite its wide use in the treatment of unresectable locally advanced or metastatic disease, a majority of patients ultimately relapsed such that overall survival was not affected [4].

For advanced STS, anthracyclines are considered the first-line therapy, with doxorubicin being prescribed most often for the systemic treatment of STS. Response rates exceeding 20% have been reported with doxorubicin alone or in combination with ifosfamide. However, the median survival of patients with metastatic STS has not improved beyond 12 months [5].

The standard second-line therapy in STS patients following failure of doxorubicin and ifosfamide is not defined. Some agents including pazopanib, gemcitabine, taxanes, trabectedin, and dacarbazine have shown promising activity. In addition, increasing evidence for “histology-tailored chemotherapy” has been observed in the last few years. However, recent evidence suggests that the combination of epirubicin and ifosfamide, regardless of the underlying histology, is superior to the selected histology-driven chemotherapy regimens [6–8]. Therefore, an understanding of the chemosensitivity of STS may result in more individualized treatment options. Recently, eribulin has been included also in an European Organization for Research and Treatment Cancer (EORTC) Phase 2 study of patients with STS and has been approved by the FDA for the treatment of liposarcomas [4, 9]. Accordingly, the quality of life (QoL) of STS patients has become an increasingly important endpoint of clinical trials for drug development and evaluation.

Several studies have been published indicating that STS and its treatment negatively impact patient health-related quality of life (HRQoL). Studies evaluating the impact of pre- and post-radiative surgery outcomes among patients with STS suggest that the magnitude of surgery-related impairment explained 54% of the decline in HRQoL, while participation restrictions (the ability to participate in activities with friends and families) explained 61% of the variation in HRQoL [10]. Other studies using the European Organization for Research and Treatment of Cancer 30-item core QoL questionnaire (EORTC QLQ-C30) indicate that disease progression is associated with a 30.26-point decline in Global Health Status [11]. When taken as a whole, these data suggest that differences between treatments on HRQoL impact are of potential utility when selecting a treatment regime for patients.

Understanding QoL/HRQoL and the detrimental impact of disease progression is critical for long-term care and survival and has become an increasingly important endpoint of clinical trials for drug development and evaluation. Therefore, QoL/HRQoL are our priority in the palliative care of all tumor patients, where curative treatment is no longer possible.

## 2. Objectives

The primary objective of this analysis was to explore the relationship between disease progression and HRQoL using patient-reported outcome data from Eisai's study E7389-G000-309 “A Randomized, Open-label, Multicenter, Phase 3 Study to Compare the Efficacy and Safety of Eribulin with Dacarbazine in Patients with Soft Tissue Sarcoma.” Specifically, the purpose was twofold: (1) to identify differences in functional outcomes and symptom severity between eribulin and dacarbazine with respect to histology and (2) to determine the extent to which disease progression is associated with changes in HRQoL among patients with advanced or metastatic STS.

## 3. Methods

### 3.1. Study Design

This was a 1 : 1 randomized, open-label, multicenter, Phase 3 study comparing the efficacy and safety of eribulin (Treatment Arm A) versus dacarbazine (Treatment Arm B) in approximately 450 patients with advanced STS (either liposarcoma or leiomyosarcoma) at approximately 110 study sites globally. The entire study consisted of three consecutive phases: Prerandomization, Randomization, and an Optional Extension. All protocol deviations were reviewed and determined prior to database lock by the study director, the study statistician, the study data manager, and the study clinical operations manager. The review was conducted in a blinded manner without looking into subject treatment code or efficacy data.

The Prerandomization phase was no longer than 21 days and included two periods: screening (Day −21 to Day −2) and baseline (Day −1). During the Prerandomization phase, patients' eligibility and baseline data including demographics (age, gender, and race/ethnicity), New York Heart Association (NYHA) functional classification, Eastern Cooperative Oncology Group (ECOG) performance status, STS-specific screening assessments (diagnosis length, STS history and tumor grade, and pathological tumor node metastasis stage at diagnosis), and past treatment history data (surgical, medical, and radiation therapy) were examined or collected.

At Randomization (Day 0), the allocation of randomization numbers was performed using an interactive voice/web response system vendor based upon the following stratification factors: (a) histology (either liposarcoma or leiomyosarcoma), (b) region [Region 1: US and Canada; Region 2: Western Europe, Australia, and Israel; or Region 3: Eastern Europe, Latin America, and Asia], and (c) number of prior treatment regimens for advanced STS (≥ 2).

**Figure 1:**
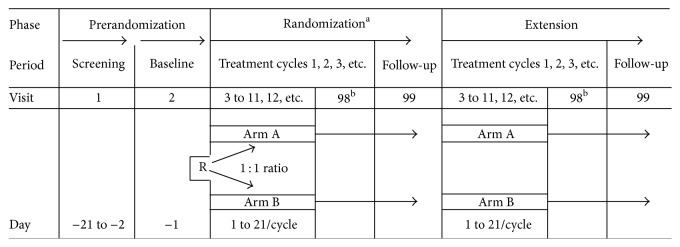
R: randomization. Arm A: eribulin mesylate 1.4 mg/m^2^ IV on Days 1 and 8, every 21 days. Arm B: dacarbazine IV on Day 1, every 21 days. The starting dose must be selected from one of the following doses: 850 mg/m^2^, 1,000 mg/m^2^, or 1,200 mg/m^2^. ^a^The randomization phase will end at the time of data cut-off for the primary analysis when the target number of events has been observed. All subjects still on treatment with study treatment or in survival follow-up will then enter the extension phase. ^b^Off-treatment visit.

The randomization and extension phases each consisted of two periods: a treatment cycle and a follow-up period. A summary of each phase is provided in Figure 1.

### 3.2. Inclusion/Exclusion Criteria

The enrolled patients with STS were not responsive to surgery and/or radiotherapy and had disease progression within 6 months of randomization. The patients had measurable disease according to the Response Evaluation Criteria in Solid Tumors version 1.1 (RECIST 1.1), with the modification that a chest X-ray could not be used for the assessment of chest lesions.

### 3.3. Analysis Populations

The analysis baseline population included all patients with available baseline data. Patients that did not meet all of the inclusion criteria or that met any of the exclusion criteria of the E7389-G000-309 clinical study were not eligible to receive study treatment. The analysis progression population included patients who met the criteria for disease progression. This schedule for tumor assessments was maintained irrespective of treatment delays.

### 3.4. Clinical Outcome Assessments

The QLQ-C30 consists of 30 questions that address five functional domains (Physical, Role, Cognitive, Emotional, and Social domains), nine symptom scales (Fatigue, Pain, Nausea and Vomiting, Dyspnea, Appetite Loss, Sleep Disturbance, Constipation, Diarrhea, and Financial Difficulties), and one global QoL scale. Items 29 (overall health) and 30 (overall QoL) are scaled from 1 “very poor” to 7 “excellent.” All scale scores are transformed to a range of 0 to 100, with higher scale scores representing a higher response level.

### 3.5. Schedule of Assessments

The QLQ-C30 was administered at baseline, on Day 1 of each treatment cycle, and at the last visit of the randomization phase. Baseline questionnaires were completed in the clinic prior to randomization. Subsequent questionnaires were completed in the clinic before any study-related procedures for that visit and before tumor assessment results were communicated to the patient. Study patients were asked to complete questionnaires at each clinic visit, even if they had declined previously. Compliance was assessed by counting completed questionnaires.

The disease progression was determined during scheduled tumor assessments and was evaluated using the RECIST 1.1 progression criteria every 6 weeks from the date of randomization during the first 12 weeks and every 9 weeks thereafter or sooner, if clinically indicated, until disease progression was confirmed by investigator histology.

The schedule for tumor assessments was maintained irrespective of treatment delays.

## 4. Statistical Methods

Quality of life analysis was conducted for both the baseline and progression populations. Two different analyses were conducted: one adjusted for histology and treatment while the other adjusted for treatment only.

For the primary analysis, results were stratified by planned treatment and histology type (leiomyosarcoma versus liposarcoma). Statistically significant differences between treatment arms were evaluated by performing a multifactor analysis of variance (ANOVA) for the progression population. The two-way ANOVA was specified using planned treatment, histology type, and their interaction term as factors. Adjusted means and standard deviation (SD) of each respective domain score were reported for the baseline population at baseline and progression population at the time of progression.

For the secondary analysis, results were stratified by planned treatment and histology type. Statistically significant differences between treatment arms were evaluated by performing an ANOVA for each population. Adjusted means and SD of each respective domain score were reported for the baseline population at baseline and for the progression population at the time of progression. For both analyses, a *p* value of less than 0.05 was considered statistically significant.

Though change from baseline to time of progression was not conducted in this analysis due to the difference in sample size, it is important to note that a change greater than 10 points is considered meaningful for all EORTC functional domains and symptom scales [12].

## 5. Results

### 5.1. Descriptive Analysis

A total of 452 patients were randomized and included in the full analysis set (228 patients in the eribulin arm and 224 patients in the dacarbazine arm). All patients were between 24 and 83 years of age (*n* = 442 patients; mean [SD] age: 55.6 [10.77] years), male (*n* = 144 [32.6%] patients), white (*n* = 322 [72.9%] patients), from the US and Canada (169 [38.2%] patients), and of NYHA class I (*n* = 274 [62.0%] patients). There were higher percentages of patients with leiomyosarcoma than those with liposarcoma in the study (68.1% leiomyosarcoma versus 31.9% liposarcoma) overall and by treatment arm (see Table 1 for further details).

### 5.2. Baseline Results

At baseline, there were no statistical differences between the two treatment arms for any of the EORTC QLQ-C30 global health score and functioning domains (*p* > 0.05). Overall, patients had better Cognitive Functioning compared to the other domains (overall mean [SD] score of 84.2 [20.43]), but worse Global Health Status (mean [SD]: 65.1 [22.20]). The other functional domains (Physical, Role, Emotional, and Social) were comparable and had overall scores that ranged from 72.5 (28.36) to 76.6 (21.57) (Table 2).

In addition, overall patients had worse Fatigue (mean [SD]: 31.7 [24.46]), Pain (mean [SD]: 28.5 [28.25]), Insomnia (mean [SD]: 26.7 [28.54]), and Financial Difficulties (mean [SD]: 25.0 [32.50]) but better Nausea and Vomiting (mean [SD]: 7.9 [16.58]) and Diarrhea (mean [SD]: 9.8 [20.83]) compared to the other domains. All other mean symptom domains (Dyspnea, Appetite Loss, and Constipation) had scores that ranged from 16.3 (24.85) to 18.6 (24.99). When stratified by treatment arm, these results were not considered statistically different (*p* > 0.05) (Table 2).

When stratified by treatment and histology, no differences were observed between liposarcoma and leiomyosarcoma groups in either eribulin or dacarbazine patients for any of the EORTC QLQ-C30 domains (Table 3).

### 5.3. Quality of Life at Disease Progression

Of the 399 patients who experienced disease progression (both with and without adjusting for histology), dacarbazine patients had significantly lower Global Health Status (*p* = 0.008) and Physical Functioning scores (*p* = 0.002) compared to patients treated with eribulin at the time of progression. In addition, patients treated with dacarbazine also had significantly worse Nausea and Vomiting (*p* = 0.001), Insomnia (*p* = 0.035), and Appetite Loss (*p* = 0.001) compared to patients treated with eribulin at the time of progression (see Table 2).

Though no analysis of change in Physical Functioning from baseline was conducted for the progression population due to the difference in sample size at baseline, it is important to note that there was a greater than 10-point decrease in Physical Functioning scores for both liposarcoma and leiomyosarcoma histology groups in the dacarbazine arm. Role Functioning scores also decreased for eribulin patients with liposarcoma histology and both liposarcoma and leiomyosarcoma histology groups in dacarbazine-treated patients. In addition, both liposarcoma and leiomyosarcoma histology groups of dacarbazine patients had differences greater than the published threshold of 10 points in Fatigue and Appetite Loss, while eribulin patients had greater than 10-point differences in Fatigue for those with leiomyosarcoma histology and Financial Difficulties for those with liposarcoma histology. Dacarbazine patients also had changes in Pain for those with leiomyosarcoma and Dyspnea for those with liposarcoma histology.

Regardless of histology, the patients had a greater than 10-point change in dacarbazine and overall scores (total population) in Role Functioning from baseline to progression. In addition, dacarbazine patients had a greater than 10-point increase in Fatigue and Appetite Loss, while in the total population (overall), there was a greater than 10-point increase in Fatigue (see Table 3).

These differences in the mean values for the given health state are greater than the published interpretation threshold of 10 points. This indicates that it is possible to observe clinically meaningful differences between health states and the observed statistical significance.

## 6. Conclusions

Disease progression appears to be a key health state for evaluating QoL in patients with sarcoma and potentially lending additional supportive information for understanding progression-free survival. Overall, this article brings statistically relevant HRQoL results during the time of disease progression between the dacarbazine and eribulin treatment arms in the Phase 3 study of advanced/metastatic sarcoma patients. Notably higher increases in symptom severity were observed among dacarbazine patients relative to patients in the eribulin treatment arm in the areas of Fatigue, Nausea and Vomiting, and Appetite Loss. Significant differences between treatment arms were also observed in the EORTC functional scales, with the patients in the eribulin treatment arm reporting significantly higher levels of Global Health Status and Physical Functioning.

Dacarbazine patients and overall (total population) scores had a greater than 10-point change in mean value in Role Functioning at progression. Dacarbazine patients also had a greater than 10-point increase in Fatigue and Appetite Loss, while, overall, there was a greater than 10-point increase in Fatigue.

When taken as a whole, the differences in the mean value for a given health state are greater than the published interpretation threshold of 10 points and indicate that it is possible to observe clinically meaningful differences between health states and the observed statistical significance as well as illustrate worsening health states observed in the dacarbazine treatment arm.

Understanding QoL in a palliative patient population is critical for appropriately addressing a patient's needs and treatment options. The shift from extending survival to delaying deterioration in patient-reported symptom, function, and HRQoL is critical and an important goal of palliative treatment. As such, evaluation and interpretation of results of studies similar to this study bring the possibility for better treatment decisions in the future for patients with (advanced) STS. The results presented in this study suggest that HRQoL is a relevant consideration when determining therapeutic pathways for patients with advanced STS and provides support for the evaluation of patient-reported outcomes at various health states such as early treatment, ongoing treatment (e.g., progression-free survival), and postprogression.

## Figures and Tables

**Table 1:** Patients' baseline characteristics of cross-sectional population for QoL analysis.

Demographic items	Eribulin (*n* = 223)	DAC (*n* = 219)	Total (*n* = 442)	*p* value^[1]^
*Age (years)*				
*n*	223	219	442	0.263
Mean (SD)	55.5 (11.09)	55.7 (10.46)	55.6 (10.77)	
Median	56.0	56.0	56.0	
Min, Max	28.0, 83.0	24.0, 83.0	24.0, 83.0	
*Gender*				
Male	65 (29.1%)	79 (36.1%)	144 (32.6%)	0.120
Female	158 (70.9%)	140 (63.9%)	298 (67.4%)	
*Race*				
White	158 (70.9%)	164 (74.9%)	322 (72.9%)	0.865
Black or African American	6 (2.7%)	6 (2.7%)	12 (2.7%)	
Japanese	1 (0.4%)	0 (0.0%)	1 (0.2%)	
Chinese	2 (0.9%)	1 (0.5%)	3 (0.7%)	
Other Asian	15 (6.7%)	15 (6.8%)	30 (6.8%)	
Native Hawaiian or other Pacific Islander	1 (0.4%)	0 (0.0%)	1 (0.2%)	
Other	6 (2.7%)	4 (1.8%)	10 (2.3%)	
Missing	33 (15.2%)	29 (13.2%)	63 (14.3%)	
*Region*				
USA and Canada	85 (38.1%)	84 (38.4%)	169 (38.2%)	0.998
Western Europe, Australasia, and Israel	104 (46.6%)	102 (46.6%)	206 (46.6%)	
Eastern Europe, Latin America, and Asia	34 (15.2%)	33 (15.1%)	67 (15.2%)	
*ECOG PS*				
*n*	223	219	442	0.661
Mean (SD)	0.5 (0.53)	0.7 (0.58)	0.6 (0.56)	
Median	1.0	1.0	1.0	
Min, Max	0.0, 2.0	0.0, 2.0	0.0, 2.0	
*NYHA*				
Class I	144 (64.6%)	130 (59.4%)	274 (62.0%)	0.353
Class II	15 (6.7%)	19 (8.7%)	34 (7.7%)	
*Prior regimens*				
Number of prior regimens for advanced STS: 2	120 (53.8%)	120 (54.8%)	240 (54.3%)	0.836
Number of prior regimens for advanced STS: >2	103 (46.2%)	99 (45.2%)	202 (45.7%)	
*Histology/cytology*				
Liposarcoma	70 (31.4%)	71 (32.4%)	140 (31.9%)	0.816
Leiomyosarcoma	153 (68.6%)	148 (67.6%)	301 (68.1%)	

CSP, cross-sectional population; DAC, dacarbazine; ECOG PS, Eastern Cooperative Oncology Group Performance Status; NYHA, New York Heart Association; SD, standard deviation; STS, soft tissue sarcoma.

Of note, of the 452 patients randomized, only 442 patients (223 patients in the eribulin treatment arm and 219 patients in the DAC treatment arm) were included in the cross-sectional population (defined as any full analysis set patient with at least one item of QLQ-C30 or EQ-5D questionnaire at the time of randomization).

^[1]^From *t*-test on continuous variable or Chi-square test on categorical variables.

**Table 2:** Adjusted mean values at baseline and progression stratified by treatment.

Domain	Time point	Eribulin Mean (SD) BL *N* = 228 PD *N* = 208	DAC Mean (SD) BL *N* = 224 PD *N* = 191	Overall Mean (SD) BL *N* = 452 PD *N* = 399	*p* value
*Global health score and functioning*					
Global Health Status	Baseline	65.2 (23.49)	64.9 (20.63)	65.1 (22.10)	0.900
	Progression	62.1 (23.32)	56.1 (21.85)	59.3 (22.81)	0.008^*∗*^
Physical Functioning	Baseline	76.6 (22.74)	76.5 (20.37)	76.6 (21.57)	0.970
	Progression	73.3 (22.69)	65.8 (26.35)	69.7 (24.77)	0.002^*∗*^
Role Functioning	Baseline	74.0 (28.70)	74.2 (25.96)	74.1 (27.35)	0.925
	Progression	65.0 (32.95)	58.7 (31.61)	62.0 (32.43)	0.054
Emotional Functioning	Baseline	75.5 (21.73)	74.0 (22.72)	74.7 (22.21)	0.482
	Progression	71.7 (26.39)	69.4 (24.08)	70.6 (25.30)	0.365
Cognitive Functioning	Baseline	84.6 (19.49)	83.9 (21.39)	84.2 (20.43)	0.731
	Progression	81.0 (23.02)	78.7 (24.79)	79.1 (23.88)	0.337
Social Functioning	Baseline	71.7 (29.97)	73.3 (26.65)	72.5 (28.36)	0.554
	Progression	68.5 (29.59)	65.4 (28.94)	67.0 (29.29)	0.283
Symptoms Domains					
Fatigue	Baseline	31.4 (25.48)	32.0 (23.42)	31.7 (24.46)	0.788
	Progression	39.8 (26.29)	44.9 (28.38)	42.3 (27.39)	0.066
Nausea and Vomiting	Baseline	7.5 (15.02)	8.2 (18.05)	7.9 (16.58)	0.687
	Progression	7.8 (14.64)	13.7 (20.40)	10.7 (17.85)	0.001^*∗*^
Pain	Baseline	26.6 (28.14)	30.5 (28.30)	28.5 (28.25)	0.149
	Progression	34.6 (29.89)	38.7 (30.87)	36.6 (30.39)	0.175
Dyspnea	Baseline	18.4 (24.89)	18.8 (25.16)	18.6 (24.99)	0.865
	Progression	22.2 (26.07)	27.4 (29.61)	24.7 (27.91)	0.064
Insomnia	Baseline	26.0 (28.02)	27.5 (29.10)	26.7 (28.54)	0.570
	Progression	26.7 (31.41)	33.2 (29.12)	29.8 (30.46)	0.035^*∗*^
Appetite Loss	Baseline	16.8 (25.71)	18.3 (28.62)	17.6 (27.17)	0.555
	Progression	19.6 (27.26)	29.5 (32.58)	24.3 (30.30)	0.001^*∗*^
Constipation	Baseline	17.6 (25.70)	15.0 (23.94)	16.3 (24.85)	0.276
	Progression	20.4 (27.36)	22.9 (28.11)	21.6 (27.72)	0.367
Diarrhea	Baseline	9.3 (19.89)	10.3 (21.79)	9.8 (20.83)	0.622
	Progression	13.4 (22.96)	10.3 (21.19)	11.9 (22.15)	0.168
Financial Difficulties	Baseline	24.3 (32.78)	25.7 (32.27)	25.0 (32.50)	0.669
	Progression	29.8 (35.87)	29.1 (32.35)	29.5 (34.19)	0.847

*∗* indicates *p* values that are less than 0.05 and are considered statistically significant. DAC: dacarbazine.

**Table 3:** Adjusted mean values at baseline and progression stratified by treatment and histology.

Domain/time point	Eribulin		DAC		*p* value
	Liposarcoma	Leiomyosarcoma	Liposarcoma	Leiomyosarcoma	
	Mean (SD) BL *N* = 71 PD *N* = 53	Mean (SD) BL *N* = 157 PD *N* = 156	Mean (SD) BL *N* = 72 PD *N* = 49	Mean (SD) BL *N* = 152 PD *N* = 142	
*Global health score and functioning*					
Global Health Status					
Baseline	64.6 (24.40)	65.5 (23.15)	64.6 (20.93)	65.1 (20.55)	—
Progression	61.3 (23.52)	62.4 (23.33)	57.5 (20.29)	55.6 (22.42)	0.008^*∗*^
Physical Functioning					
Baseline	73.2 (25.76)	78.2 (21.12)	76.8 (21.53)	76.4 (19.87)	—
Progression	71.4 (24.33)	74.0 (22.15)	63.9 (28.93)	66.4 (25.48)	0.002^*∗*^
Role Functioning					
Baseline	69.8 (31.61)	75.9 (27.18)	75.0 (26.28)	73.9 (25.89)	—
Progression	57.9 (36.63)	67.4 (31.34)	60.2 (29.82)	58.2 (32.30)	0.054
Emotional Functioning					
Baseline	73.1 (23.10)	76.6 (21.05)	73.0 (24.51)	74.4 (21.88)	—
Progression	73.1 (27.33)	71.2 (26.13)	69.9 (23.50)	69.2 (24.40)	0.366
Cognitive Functioning					
Baseline	84.8 (19.26)	84.4 (19.66)	83.1 (21.83)	84.2 (21.24)	—
Progression	82.4 (20.26)	80.5 (23.94)	77.6 (24.90)	79.1 (24.82)	0.338
Social Functioning					
Baseline	64.7 (33.52)	74.8 (27.78)	71.8 (27.69)	74.0 (26.20)	—
Progression	62.3 (35.98)	70.6 (26.87)	63.2 (29.06)	66.1 (28.96)	0.282
*Symptom scales*					
Fatigue					
Baseline	34.1 (27.44)	30.1 (24.53)	32.6 (25.98)	31.7 (22.14)	—
Progression	39.2 (28.46)	40.1 (25.61)	43.5 (28.03)	45.4 (28.58)	0.067
Nausea and Vomiting					
Baseline	8.2 (17.30)	7.2 (13.92)	8.9 (18.00)	7.8 (18.12)	—
Progression	7.2 (11.09)	8.1 (15.70)	12.9 (22.12)	14.1 (19.85)	0.001^*∗*^
Pain					
Baseline	28.3 (31.09)	25.9 (26.77)	31.9 (26.88)	29.9 (29.03)	—
Progression	35.8 (30.03)	34.2 (29.92)	34.4 (24.86)	40.3 (32.63)	0.175
Dyspnea					
Baseline	16.9 (22.60)	19.1 (25.91)	19.2 (27.98)	18.6 (23.77)	—
Progression	22.0 (25.27)	22.3 (26.42)	35.4 (33.62)	24.6 (27.70)	0.063
Insomnia					
Baseline	24.2 (29.64)	26.8 (27.32)	24.4 (28.71)	29.0 (29.27)	—
Progression	30.8 (33.56)	25.4 (30.65)	28.6 (27.22)	34.7 (29.67)	0.035^*∗*^
Appetite Loss					
Baseline	16.9 (25.96)	16.8 (25.68)	18.3 (29.70)	18.4 (28.18)	—
Progression	18.2 (24.95)	20.0 (28.07)	29.9 (32.09)	29.3 (32.85)	0.001^*∗*^
Constipation					
Baseline	19.8 (30.42)	16.6 (23.30)	17.4 (26.94)	13.8 (22.36)	—
Progression	20.7 (25.51)	20.2 (28.04)	23.8 (27.22)	22.5 (28.50)	0.368
Diarrhea					
Baseline	10.6 (21.76)	8.7 (19.03)	12.2 (23.39)	9.4 (20.99)	—
Progression	13.8 (18.98)	13.2 (24.23)	11.6 (25.05)	9.9 (19.77)	0.169
Financial Difficulties					
Baseline	26.1 (34.24)	23.5 (32.19)	25.4 (33.08)	25.8 (31.99)	—
Progression	37.1 (42.70)	27.3 (33.00)	34.7 (33.99)	27.2 (31.67)	0.846

*∗* indicates *p* values that are less than 0.05 and are considered statistically significant. DAC: dacarbazine.
